# Alternative approaches to myeloid suppressor cell therapy in transplantation: comparing regulatory macrophages to tolerogenic DCs and MDSCs

**DOI:** 10.1186/2047-1440-1-17

**Published:** 2012-09-28

**Authors:** Paloma Riquelme, Edward K Geissler, James A Hutchinson

**Affiliations:** 1 Department of Surgery, University Hospital Regensburg, Franz-Josef-Strauss-Allee 11, Regensburg, 93053, Germany

**Keywords:** Regulatory macrophage, M reg, Cell-based medicinal product, The ONE Study

## Abstract

Several types of myeloid suppressor cell are currently being developed as cell-based immunosuppressive agents. Despite detailed knowledge about the molecular and cellular functions of these cell types, expert opinions differ on how to best implement such therapies in solid organ transplantation. Efforts in our laboratory to develop a cell-based medicinal product for promoting tolerance in renal transplant patients have focused on a type of suppressor macrophage, which we call the *regulatory macrophage* (M reg). Our favoured clinical strategy is to administer donor-derived M regs to recipients one week prior to transplantation. In contrast, many groups working with tolerogenic dendritic cells (DCs) advocate post-transplant administration of recipient-derived cells. A third alternative, using myeloid-derived suppressor cells, presumably demands that cells are given around the time of transplantation, so that they can infiltrate the graft to create a suppressive environment. On present evidence, it is not possible to say which cell type and treatment strategy might be clinically superior. This review seeks to position our basic scientific and early-stage clinical studies of human regulatory macrophages within the broader context of myeloid suppressor cell therapy in transplantation.

## Introduction

The existence of anti-inflammatory T cell-suppressive cells of the myeloid lineage has long been recognised and the ability of such cells to induce tolerance to auto- and allo-antigens after adoptive transfer has been studied extensively. Although often mooted, progress towards clinical applications of myeloid suppressor cell therapy was limited until recently, when several independent groups began trials in transplantation [1-3], rheumatoid arthritis [4] and diabetes [5]. Results from these early-phase clinical studies have been promising, at least in terms of demonstrating the feasibility and risk profile of such approaches, but evidence of efficacy in patients is still lacking. In this regard, the inception of *The ONE Study*, a clinical trial of cell therapy as an adjunct immunosuppressive treatment in renal transplantation, is a critical step forward [6].

Currently, the concept of promoting immunological regulation in transplant recipients by treating with myeloid suppressor cells of various types is being pursued by a number of groups [7-9]. Depending upon their exact nature and whether they are of donor, recipient or third-party origin, different myeloid suppressor cells exert their therapeutic effects through very different mechanisms [10]. In turn, the immunological actions of particular myeloid suppressor cells determine how those cells might be optimally delivered to patients, especially with respect to timing and route of administration, and immunosuppressive co-treatments. This review examines three radically alternative approaches to myeloid suppressor cell therapy in transplantation, each with its own clinical and immunological merits.

Broadly speaking, myeloid suppressor cells are characterised either by an arrested state of immaturity, when they are known as *myeloid-derived suppressor cells* (MDSCs) or *tolerogenic DCs*, or by a more mature phenotype, reflecting the ability of myeloid antigen-presenting cells to switch into a suppressive mode under certain conditions (  1). Diverse anti-inflammatory treatments prevent DC maturation *in vitro*, including generation in the presence of IL-10 (DC-10) [11] or rapamycin (Rapa-DC) [12], culture in low concentrations of GM-CSF (Tol-DC)[13] or exposure to dexamethasone and vitamin D [4]. Paradoxically, various pro-inflammatory factors can also drive macrophages and DCs to a suppressive state, including IFN-γ, prostaglandin E_2_ (PGE_2_) and repetitive Toll-like Receptor (TLR) stimulation. As we describe below, the *regulatory macrophage* (M reg) is an important example of an activation-induced myeloid suppressor cell.

**Figure 1:**
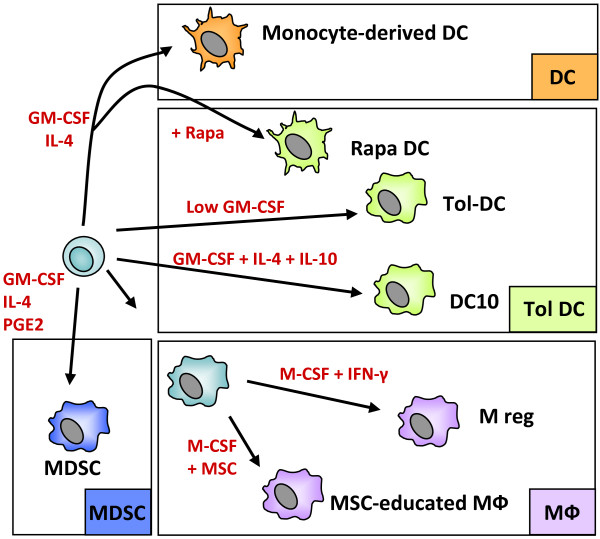
**The spectrum of monocyte-derived suppressor APCs.** Suppressor macrophages and DCs can be generated from monocytes using M-CSF or GM-CSF, with or without IL-4. Development of immature DCs into mature, activating DCs can be blocked by various substances, including rapamycin or dexamethasone and vitamin D. DCs can also be rendered tolerogenic by culture in low-dose GM-CSF or by addition of suppressive cytokines, such as IL-10 or TGF-β1. Mesenchymal stem cells (MSC) can induce a suppressor phenotype in co-cultured macrophages. Myeloid-derived suppressor cells (MDSC) can be generated by exposing monocytes/macrophages to tumour-secreted factors, most notably PGE_2_.

It is not known whether the distinction between myeloid suppressor cells in a state of arrested immaturity and those in an activation-induced suppressor state is biologically meaningful. Certainly, many of the same molecular mechanisms account for the suppressive activities of both immature and activation-induced myeloid suppressor cells (Table 1). It is also unclear whether the various types of tolerogenic DCs and MDSCs described in the literature represent unique cell subtypes, or whether they are functionally interchangeable cells with only superficial phenotypic differences. A workshop recently convened by *The ONE Study* consortium in Regensburg, Germany, should provide some insight into these unresolved issues (see accompanying editorial [6]).

**Table 1:** Main characteristics of different myeloid suppressor APCs

	**Human phenotype**	**Function**	**Mechanism**	**Trafficking**	**Ref.**
M reg	CD14^-/low^, CD16^-^, CD80^-/low^, CD40^-/low^, HLA-DR^+^, TLR2^-^, CD83^-^, CD163^-/low^	T cell elimination, suppression of T cell proliferation	IDO	Lung, blood, liver, spleen	[3,14-16]
Tol-DC	HLA-DR^+^, low costimulation, CD14^+^, CD11b^+^, resistant to maturation	Allo-Ag capture and presentation, suppression of T cell proliferation	HO-1, EBl3, iNOS	Spleen	[8,17-20]
DC-10	CD14^+^, CD16^+^, CD83^+^, CD1a-, ILT4^+^, HLA-G^+^, IL-12^-^	Induction T cell anergy, Tr-1 cell induction	IL-10, ILT-4-HLA-G interactions	Blood and secondary lymphoid organs	[21]
Rapa-DC	HLA-DR^+^, low costimulation, resistant to maturation, IL-12^+^	T cell hyporesponsiveness and apoptosis, T reg induction	Low costimulation	LN, spleen	[22-26]
MDSC	HLA-DR^-/low^, CD11b^++^, CD14^+^, CD33^+^, CD34^+^	Suppression of T cell proliferation, cytokine production, apoptosis in T cells	iNOS, Arg-1, ROS, PGE_2_, HO-1, TGF-β, cys depletion	Blood, graft, spleen, LN	[27-32]

### Regulatory macrophages

#### Human regulatory macrophages

Efforts in our laboratory to develop a cell-based medicinal product for use in promoting transplant tolerance in renal transplant patients have focussed on M regs. The human M reg reflects a unique state of macrophage differentiation, distinguished from macrophages in other activation states by its particular mode of derivation, robust phenotype and potent T cell suppressor function. These cells arise from CD14^+^ peripheral blood monocytes during a seven-day culture period during which the cells are exposed to M-CSF, 10% human serum and a final 24-hour pulse of IFN-γ [14]. M regs derived in this manner adopt a characteristic morphology and are homogeneously CD14^-/low^ HLA-DR^+^ CD80^-/low^ CD86^+^ CD16^-^ CD64^+^ TLR2^-^ TLR4^-^ and CD163^-/low^. M regs do not stimulate allogeneic T cell proliferation *in vitro* and, when co-cultured with polyclonally stimulated T cells, are potently suppressive of proliferation. The suppressive capacity of M regs has been attributed to IFN-γ-induced indoleamine 2,3-dioxygenase (IDO) activity, as well as contact-dependent deletion of activated T cells [3]. Critically, human M regs are relatively resistant to maturation upon stimulation with lipopolysaccharide (LPS), possibly as a consequence of TLR down-regulation.

In order to assess their pattern of trafficking after central venous infusion, allogeneic M regs labelled with ^111^Indium-oxine were administered to a single patient, MM, whose case is described below [3]. Subsequently, the anatomical distribution of the M regs was tracked over 30 hours in serial whole-body Single Photon Emission Computed Tomography (SPECT) studies. Initially, M regs were only detected in the lungs, but within 2.5 hours were found circulating in blood. By 30 hours post-infusion, most M regs had emigrated from the lungs to the spleen, liver and haematopoietically-active bone marrow. M regs did not accumulate in lymph nodes. We can be confident that the majority of infused M regs survived for the duration of the follow-up, because tracer was not observed in the urinary tract or blood.

### *Mouse regulatory macrophages*

Mouse CD11b^+^ Ly6C^+^ bone marrow monocytes cultured under conditions analogous to those used in the generation of human M regs give rise to a population of suppressive macrophages which are highly similar to human M regs in morphology, cell-surface phenotype and *in vitro* function [16]. Mouse M regs express a selection of typical macrophage markers, including CD11b, CD11c, CD68, F4/80 and CD14, and exhibit a partially-matured phenotype with intermediate levels of MHC Class II and CD80, and no expression of CD40 or CD86. Mouse M regs express sialoadhesin (CD169), macrophage scavenger receptor (CD204) and Dectin-1, but lack other markers of notable tissue macrophage subsets, such as Dectin-2, MARCO, CD4, CD206 and CD209. M regs do not express Ly6C or Ly6G, which together constitute the Gr-1 antigen that is expressed by all mouse MDSCs. CD11c is homogenously expressed by mouse M regs, but they do not express other DC subset-defining markers, including 33D1, OX40L (CD252), CD103, CD205 and CD207. Importantly, like human M regs, mouse M regs do not express TLR2 or TLR4. Given its mode of derivation, morphology and cell-surface phenotype, it seems most appropriate to classify the M reg as a macrophage; however, mouse M regs do not express markers typical of either M1-polarised macrophages (eg. TNF-α, IL-6 and IL-12b) or M2-polarised macrophages (eg. CD206, Ym1 or Fizz1). To better understand the phenotypic relationship between mouse M regs and previously-described macrophage polarisation states, we performed whole-genome gene expression studies to compare M regs to monocytes, monocyte-derived DCs, resting macrophages, IFN-γ-stimulated macrophages and M1-, M2a-, M2b- and M2c-polarised macrophages: These experiments showed that mouse M regs represent a novel and unique state of macrophage activation. Mouse M regs inhibit T cell responses *in vitro* by several mechanisms. Mitogen-stimulated T cell proliferation is non-specifically inhibited in co-cultures with M regs through the action of inducible nitric oxide synthase (iNOS). M regs delete co-cultured allogeneic T cells (but not isogeneic T cells) through an unknown mechanism that ultimately leads to phagocytosis of the T cells. Any T cells which survive direct co-culture with M regs are impaired in their ability to secrete IL-2 and IFN-γ upon specific and non-specific restimulation [16].

Preclinical experiments using a heterotopic mouse heart transplant model demonstrate the potential of mouse M regs to prolong allograft survival [16]. A single intravenous administration of 5×10^6^ donor-strain M regs at 8 days prior to transplantation significantly prolongs allograft survival in unconditioned, non-immunosuppressed recipients using both the stringent C3H-to-BALB/c (32.6 ± 4.5 vs. 8.7 ± 0.2 days) and B6-to-BALB/c (31.1 ± 12 vs 9.7 ± 0.4 days) strain combinations. This graft-protective effect is specific to donor cells, as recipient cells do not prolong graft survival compared to untreated controls and third party-derived M regs provide only a marginal benefit (11.0 ± 0.6 days). Improved graft survival is observed irrespective of whether M regs are administered 8 or 35 days prior to transplantation. Co-treatment with M regs and 1 mg/kg/day rapamycin for 10 days post-transplant enhances the graft-protective effect of M regs (64.1 ± 8.6 days) compared to treatment with M regs alone or rapamycin alone, and some recipients co-treated with M regs and rapamycin accept their allografts indefinitely. The mechanism of M reg-mediated allograft protection is iNOS-dependent because M regs derived from Nos2-deficient mice only marginally prolong graft survival (12.0 ± 1.8 days). Very importantly, the iNOS-dependence of M reg treatment *in vivo* proves that the graft-protective effect of M regs is not simply due to alloantigen exposure, but must be mediated by living, metabolically-competent cells.

**Figure 2:**
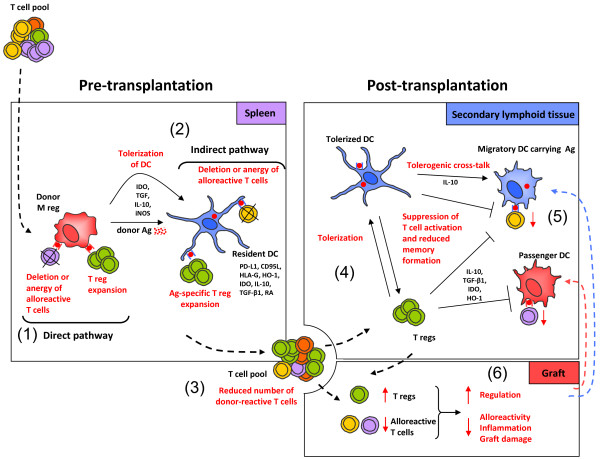
**Proposed mode of M reg action.** (**1**) When administered prior to transplantation, donor M regs migrate to spleen, where they present donor antigen through the direct pathway to alloreactive T cells and either delete or anergise them, or induce expansion of regulatory T cells. Human M regs have been shown to delete activated T cells through a contact-dependent mechanism and to suppress T cell proliferation through IDO; however, other suppressor mechanisms may also contribute to M reg function, such as IL-10 and TGF-β secretion, or iNOS activity. (**2**) It is likely that M regs serve as a source of donor alloantigen, which is captured and presented by immature recipient DCs to alloreactive T cells via the indirect and semi-direct pathways of alloantigen recognition. In consequence, responding T cells may be deleted or anergised, and antigen-specific T regs may be induced. (**3**) Through these mechanisms, the recipient T cell pool is enriched for T regs and depleted of donor-reactive T cells. (**4**) After transplantation, recipient T regs could induce tolerogenic DCs in secondary lymphoid organs. (**5**) Recipient tolerogenic DCs could then suppress activation of T cells. (**6**) In consequence, an immunological environment conducive to allograft acceptance is established.

Several mutually redundant mechanisms might be invoked to explain the effects of M regs *in vivo* and, in our opinion, more than one mechanism is likely to be in operation (Figure 2). It is well-known that pre-transplant exposure to donor alloantigen promotes allograft acceptance [33] and that delivery of alloantigen as apototic cell debris enhances this effect [34,35]. Both CD8α^+^ DCs [36] and F4/80^+^ PD-L1^+^ IL-10-producing macrophages [37] of the splenic marginal zone appear to be important for the tolerogenic effects of complement-opsonised apoptotic antigens [38]. After intravenous injection into mice, isogeneic and allogeneic mouse M regs are initially trapped in the pulmonary vasculature, then rapidly redistribute to other peripheral organs, especially the liver and spleen, but not to lymph nodes [16]. Isogeneic and allogeneic M regs are relatively short-lived after transfer into immunocompetent mice, both being detectable at 2 weeks post-infusion, but not 4 weeks. These experiments suggest that M regs have an inherently limited lifespan after transfer; therefore, M regs probably serve as a source of apoptotic donor alloantigen-expressing material. Such a mechanism operates in tolerance induction protocols using donor-specific transfusion (DST) and αCD154 treatment, in which indirect presentation of donor alloantigen results in a predominantly deletional tolerance [39]. And yet, the graft-protective effect of donor alloantigen exposure in the absence of costimulatory blockade or lymphodepletive conditioning [40] is rarely as profound as that achieved with M reg treatment, even in less stringent transplant models. Moreover, it is difficult to reconcile the requirement for iNOS expression by transferred M regs with the suggestion that M regs act merely as a passive source of alloantigen. It is possible that mouse M regs directly suppress T cell responses *in vivo* through iNOS activity, as they do *in vitro*, but the critical action of iNOS might equally be mediated by recipient APCs [41,42]. Accordingly, one important fate of M regs might be to migrate into tissues, induce a suppressive condition in recipient APCs, before dying in a suitably self-conditioned environment.

### MDSCs and tolerogenic DCs

Naturally-occuring myeloid suppressor cells fall into three ‘classes’: those present in non-inflamed tissues; those arising in inflamed tissues; and dedicated myeloid suppressor cell populations which are recruited to both inflamed and non-inflamed tissues. The ‘default’ condition of immature DCs and macrophages in non-inflamed tissues is suppressive; such cells play crucial roles in the maintenance of tissue homeostasis and self-tolerance, as well as resisting the otherwise perpetual activation of inflammatory responses [43]. Suppressive macrophages and DCs can also arise in inflamed tissues through conversion from activated, mature APCs; such cells limit the extent of inflammatory responses and promote tissue-reparative processes and the re-establishment of tissue homeostasis [44]. Committed myeloid suppressor cell populations, collectively known as MDSCs, are present in blood and can infiltrate both inflamed and non-inflamed tissues, including allografts and tumours [45]. These three general classes of naturally-occurring myeloid suppressor cell are reflected by the types of myeloid suppressor cell that can be generated *in vitro*.

Immature macrophages and DCs are normal stromal components of almost all peripheral tissues, where they serve many vital functions, including the removal of dead cells, microbial products and other tissue debris by phagocytosis, and the maintenance of a non-inflammatory environment through secretion of suppressive mediators. The immature state of tissue-resident macrophages and DCs is stabilised by anti-inflammatory factors present in non-inflamed tissues, such as IL-10 and glucocorticoid. Colonic macrophages exemplify the role of immature, tissue-resident APCs in preventing aberrant inflammatory responses; these cells respond to IL-10 by secreting IL-10, which suppresses the continual inflammation that would otherwise be caused by gut commensals and their products [46]. Besides their local effects in tissues, immature DCs deliver self-antigens to secondary lymphoid organs, where they are presented to T cells in a poor co-stimulatory context to propagate self-tolerance. Many *in vitro*-derived tolerogenic DCs fit this description of a phagocytic, maturation-resistant cell with the ability to present antigen in the absence of strong co-stimulation, including DC-10, Rapa-DC and Tol-DC [10]. A wide range of unrelated stimuli drive DCs to suppressive states, so there is no unique phenotype or mechanism of action associated with tolerogenic DC populations (Table 1). Generically, human tolerogenic DCs express cell-surface markers typical of immature monocyte-derived DCs, but there are markers of specific subtypes: Tol-DC are CD11c^+^ CD11b^+^ EBI3^+^ cells; DC-10 express high levels of HLA-G, ILT4 and secrete IL-10; Rapa-DC lack expression of CD80 and CD86, express only low-levels of HLA-DR, but secrete significant amounts of IL-12. These three tolerogenic DC subtypes exert their suppressive effects through alternative mechanisms, specifically: Tol-DC inhibit T cell responses through haemoxygenase (HO)-1 activity and T reg expansion [17]; DC-10 suppress T cell proliferation and induce antigen-specific Tr-1 cells through IL-10 production [21]; Rapa-DC induce effector T cell anergy and promote FoxP3^+^ T reg generation by presenting antigen in the absence of costimulation. Tolerogenic DC populations also differ in their migratory capacity *in vivo*. Studies in animals have shown that Rapa-DCs traffic to lymph nodes [25,26], but that Tol-DCs migrate preferentially to spleen [8,20].

In response to tissue injury or detection of pathogens, tissue-resident macrophages and DCs initiate a localised inflammatory response, involving the recruitment of other immunological effector cells, resulting in secondary tissue damage. However, this destructive behaviour is usually only transient, because activated macrophages and DCs switch to an anti-inflammatory and tissue-reparative mode [44]. The ability of macrophages to both exacerbate and attenuate inflammatory reactions is neatly illustrated by their contribution to ischaemia-reperfusion injury and its resolution: Experimental depletion of macrophages prior to renal ischaemia-reperfusion injury reduces the number of infiltrating leucocytes and helps to preserve short-term kidney function [47]; however, the cost of preventing early macrophage-mediated injury is a worse tissue repair response and impaired long-term function [48]. We regard human M regs, which are activated during cell culture by adherence to plastic, serum components and IFN-γ, as belonging to the class of activation-induced (or ‘deactivated’) myeloid suppressor cells.

MDSCs represent a third class of myeloid suppressor cell, which is characterised by the expression of markers associated with myeloid progenitors and commitment to a suppressor phenotype prior to entering tissues from blood. Although MDSC populations in mice and humans are highly heterogenous, any standard definition of mouse MDSCs includes expression of CD11b and Gr-1, whereas human MDSCs universally express CD11b, CD33, CD34 and VEGFR1 [45]. It is well-established that MDSCs exert a local immunosuppressive effect within solid tumours [49,50] and it appears that they have a similar function in transplanted organs, because induction of tolerance to kidney, skin and cardiac allografts is associated with infiltration of grafts by MDSCs [28,51].

### Alternative clinical approaches

In principle, myeloid suppressor cells could favour allograft acceptance in several ways. Firstly, myeloid suppressor cells could exert transient, general immunosuppressive effects by secreting anti-inflammatory mediators or releasing of apoptotic debris. Secondly, myeloid suppressor cells could suppress inflammation and promote tissue repair processes within allografts during the immediate postoperative period. Thirdly, myeloid suppressor cells could anergize or delete recipient effector T cells, or induce alloantigen-specific regulatory T cells. Intuitively, the relative contribution of these allograft-protective mechanisms to transplant survival will depend on the route and timing of therapeutic cell administration, and the type of myeloid suppressor cell being used.

### *Pre-transplant versus post-transplant cell administration*

Arguably, the state of the immune system prior to transplantation is more conducive to the establishment of tolerance than afterwards. Under steady-state, physiological conditions, immature DCs capture and present innocuous antigens leading to the anergy or deletion of antigen-reactive effector T cells and the expansion of specific T regs [9]. Organ transplantation disrupts this homeostatic condition by causing massive inflammation and the abrupt activation of vast numbers of alloreactive recipient T cells. Hence, the rationale for using myeloid suppressor cells prior to transplantation is that enrichment of alloantigen-specific T regs and deletion of effector T cells should be easier in an immunological environment naturally predisposed to the maintenance of tolerance. In addition, conventional immunosuppressive therapy might antagonise the regulatory action of myeloid suppressor cells given after transplantation.

Whilst exploiting the pro-tolerogenic condition of the pre-transplant immune system seems a sensible therapeutic strategy, a few arguments have been levelled against this approach. Firstly, and most importantly, in order to induce antigen-specific immunological regulation prior to transplantation, it is necessary to deliberately expose the recipient to donor alloantigen, which carries an attendant risk of allo-sensitisation. Secondly, patients with end-stage organ failure awaiting transplantation may not be in a immunologically quiescent state, either because of their underlying disease or concurrent subclinical infections. Thirdly, pre-treatment with donor-derived myeloid suppressor cells is not possible in the case of transplantation from deceased donors.

### *Myeloid suppressor cells of donor versus recipient origin*

From an immunological standpoint, the principal reason for using myeloid suppressor cells of donor origin is to expose the recipient to intact donor alloantigen via the direct and semi-direct pathways. Alloantigen released by donor-derived cells could also be captured and presented in a pro-tolerogenic context by immature recipient DCs via the indirect pathway [52]. Consequently, donor-derived myeloid suppressor cells find their main application in pre-transplant conditioning therapies [3,53]. Donor-derived cells have the advantage that they can be reliably obtained from healthy, living donors. Recipient-derived myeloid suppressor cells are less likely to be eliminated by recipient T cells and NK cells, so have a greater capacity to migrate and engraft, and are less likely to sensitise the recipient against donor alloantigen. Production of recipient-derived myeloid suppressor cells prior to deceased-donor transplantation is feasible; however, since recipient-derived myeloid suppressor cells must capture and present graft-derived alloantigen in the indirect pathway, postoperative administration is the generally favoured approach of groups working with recipient-derived cells. Recipient-derived myeloid suppressor cells can be loaded with donor-alloantigen prior to infusion and this approach has proven to be a very effective therapy in animal models. Notably, antigen-pulsed tolerogenic DC were found to induce transplantation tolerance by expanding T regs which recognised alloantigen in the indirect pathway [25]. It has also been suggested that using third-party myeloid suppressor cells could eliminate some of the risks inherent to using donor or recipients cells, although it is difficult to envisage how third-party cells could induce allo-specific regulation, especially considering that the use of third-party tolerogenic DCs shows little or no effect in many animal models [20,25,54].

### *Route of administration*

Selecting a route of administration for a tolerogenic cell therapy involves a trade-off between the most efficient means of delivering cells to their site of action and issues of clinical practicality and safety. Most animal experiments with myeloid suppressor cells have evaluated their therapeutic potential after intravenous administration [16,20,25], although some groups have investigated subcutaneous [55] and intramuscular injection [26]. The intravenous route seems quite suitable for M regs, since they normally traffic to liver, spleen and bone marrow. Tol-DCs and Rapa-DCs prolong allograft survival after intravenous injection; however, because recipient-derived tolerogenic DCs must capture graft antigens and suppress T cell activation in graft-draining lymph nodes, direct application of cells into lymph nodes may prove a superior route of administration. Since MDSCs exert important immunosuppressive actions within allografts, the possibility of injecting them directly into the arterial supply of the transplanted organ deserves further investigation.

### Clinical applications of M reg therapy

As we have seen, when applying myeloid suppressor cell therapy in solid organ transplantation, the choice of myeloid suppressor cell type, whether it is given pre- or post-transplantation, whether it is of donor or recipient origin, and its route of administration are interdependent considerations (Table 2).

**Table 2:** Clinical translation of myeloid suppressor cell therapy in solid organ transplantation

	**Origin**	**Time**	**Pre-clinical**	**Clinical**	**Ref.**
M reg	Donor	Pre-	Mouse, pig, dog	TAIC- I, TAIC-II, The ONE Study	[1-3,6,16,56-58]
Tol-DC	Recipient	Peri-/post-	Rat, NHP	The ONE Study	[6,18,20,53,54]
DC-10	Recipient/Donor DC10 + recipient Tr-1	Peri-/post-	Rat	-	[59]
Rapa-DC	Donor-pulsed recipient	Pre-Post	Mouse	-	[25]
MDSC	Recipient	Peri-/Post-	Mouse	-	[60]

On the present evidence, we cannot say which cell type or clinical approach represents an optimal therapy; however, based on our preclinical animal studies and the outcomes of the TAIC-I and TAIC-II clinical trials, our research group favours the preoperative administration of donor-derived M regs. A handful of clinical trials have been conducted (or are presently underway) using tolerogenic DCs in the treatment of Type I diabetes [5] and rheumatoid arthritis [4]; however, these cells have not yet been applied in solid organ transplantation. As we describe below, M reg-containing cell preparations have now been trialled in a total of 21 renal transplant recipients (Table 3).

**Table 3:** Clinical studies with regulatory macrophages

**Study**	**n**	**Source**	**Time**	**Total cell number**	**Cells/kg body weight**	**Ref.**
KW	1	Donor spleen	+5		1.1 × 10^6^	[56]
TAIC-I	12	Donor spleen	+5	0.9-5 × 10^8^	1-7.5 × 10^6^	[1]
FR	1	Donor blood	−17	4.8 × 10^9^	6.9 × 10^7^	[57]
TAIC-II	5	Donor blood	−5	1.4-5.9 × 10^9^	1.7-10.4 × 10^7^	[2]
M reg: MM, CA	2	Donor blood	−6/7	4.3-7.5 × 10^8^	7-8 × 10^6^	[3]

### *The TAIC-I clinical trial*

**Figure 3:**
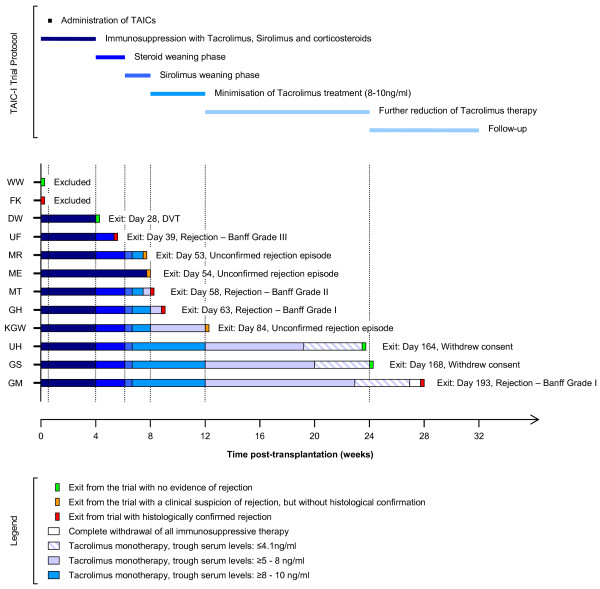
**Overview of the TAIC-I trial.** Patients enrolled in the TAIC-I Study each received a kidney transplant from a deceased donor. The mean age of the patients was 46.3 years and 9/12 patients were male. The median HLA-A,-B and –DR mismatch was 5/6. Initially, patients were treated with a combination of tacrolimus (trough levels of 10–15 ng/ml), sirolimus (trough levels of 4–8 ng/ml) and corticosteroids. Cells were infused on day 5 post-transplant. Steroids were tapered in weeks 5 and 6. Sirolimus was withdrawn in weeks 7 and 8. If graft function remained stable, tacrolimus treatment was first minimised to trough tacrolimus levels of 8–10 ng/ml by week 12 and then to levels of 5–8 ng/ml by week 24. Further reductions in tacrolimus therapy were undertaken in patients with stable graft function and no histological evidence of rejection. Figure reproduced with permission from Hutchinson, JA. et al. *Transplant International* (2008) 21:728–741.

The TAIC-I trial was a single-centre, open-label, single-arm study with the objective of obtaining information on the safety and tolerability of administering M reg-containing cell preparations to renal transplant recipients (http://www.clinicaltrials.gov, NCT00223093) [1]. A total of 12 patients receiving their first transplant from a deceased donor were enrolled in the study. Initially immunosuppression comprised tacrolimus, sirolimus and corticosteroids. From week 4 posttransplantation, patients were aggressively weaned from immunosuppressive therapy with the intention of achieving tacrolimus monotherapy (8–10 ng/ml trough level) by week 12 and further reduction to ≤4 ng/ml within 24 weeks. Patients were treated with 0.9–5.0 × 10^8^ donor-derived cells by central venous infusion at 5 days after transplantation (Figure 3). No acute complications or later adverse reactions relating to the cell infusion were observed. Thus, the TAIC-I trial demonstrated the clinical feasibility of producing and administering M reg-containing cell preparations to kidney transplant recipients.

### *The TAIC-II clinical trial*

**Figure 4:**
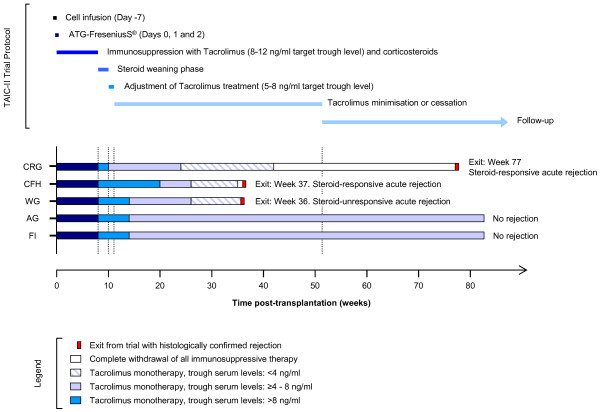
**Overview of the TAIC-II trial.** Patients enrolled in the TAIC-II Study each received a kidney transplant from a living donor. The mean age of the patients was 35.4 years and 4/5 patients were male. The median HLA-A,-B and –DR mismatch was 3/6. Cells were infused 5 days prior to transplantation. All patients received ATG induction therapy on days 0, 1 and 2. Initial maintenance immunosuppression comprised glucocorticoids and tacrolimus (8–12 ng/ml trough levels). Steroid therapy was withdrawn by week 10. Tacrolimus dosing was then adjusted into a target range of 5 – 8 ng/ml trough levels. From week 24 onwards, further reductions were made in tacrolimus monotherapy, leading to complete drug withdrawal in two patients.

The TAIC-II study was a phase I/II clinical trial (http://www.clinicaltrials.gov, NCT00223067) designed to assess the safety and efficacy of administering donor-derived M reg-containing cell preparations to recipients of living-donor renal transplants [2]. Five days prior to surgery, five living-related kidney transplant recipients were treated with 1.4-5.9 × 10^8^ cells (Figure 4). No clinical complications of the cell infusion were observed. All patients received induction therapy with anti-thymocyte globulin (ATG) on days 0, 1 and 2. From the time of transplantation onwards, patients received a dual immunosuppressive regime comprising conventional steroid treatment and tacrolimus therapy, aiming for trough levels of 8–12 ng/ml. Steroids were weaned by week 8 post-transplantation and tacrolimus was reduced to 5–8 ng/ml over several weeks. Four patients were successfully minimised to low-dose tacrolimus monotherapy. No rejection occurred in two of five patients. Following the reduction of tacrolimus treatment to <2 ng/ml for 6 weeks, one patient underwent a rejection episode at 36 weeks. The two remaining patients experienced acute rejection episodes only after complete cessation of immunosuppression for 2 and 34 weeks. All patients in TAIC-II were monitored for indices of graft acceptance and rejection through the RISET network. None of the patients developed anti-donor HLA antibodies as a consequence of M reg administration and no accelerated graft loss occurred. Anti-donor T cell reactivity was serially assayed by MLR and was found to be consistently reduced.

### *Patients MM and CA*

Since the TAIC-I and TAIC-II clinical trials, we have arrived at a detailed understanding of the derivation, phenotype and T cell-suppressive functions of *in vitro*-derived human regulatory macrophages. This knowledge has inspired methodological advances in regulatory macrophage manufacture, leading to a far purer and more homogeneous cell product, which has now been applied to two further living-donor renal transplant recipients with encouraging results [3].

**Figure 5:**
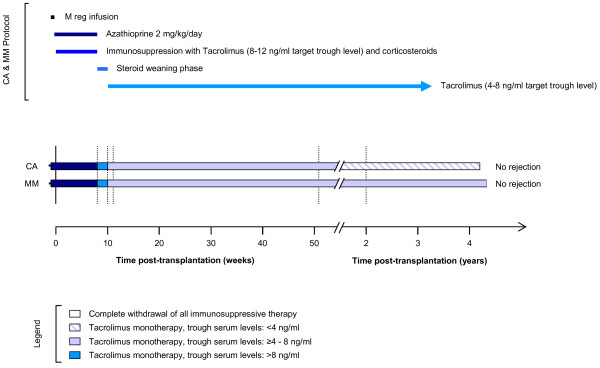
**Overview of the treatment of patients MM and CA.** Both patients received a living-donor kidney transplant. M regs were infused 6 (MM) or 7 (CA) days prior to transplantation under cover of 2 mg/kg/day azathioprine. Initial maintenance immunosuppression comprised glucocorticoids and tacrolimus (>8 ng/ml trough levels). Steroid therapy was withdrawn by week 10. Tacrolimus dosing was then adjusted into a target range of 4 – 8 ng/ml trough levels.

The first of these patients, MM, a 23 year-old female with renal failure owing to IgA nephropathy, received a living-donor kidney transplant from her 58 year-old mother. Mother and daughter had only single HLA-B and- DR mismatches (Figure 5). Six days prior to transplantation, patient MM received 8.0 × 10^6^ donor-derived M regs/kg by slow central venous infusion under cover of 2 mg/kg/d azathioprine. Conventional treatment with steroids and tacrolimus was started at the time of transplantation. Azathioprine was stopped at 8 weeks post-transplant and steroids were weaned by 14 weeks. Thereafter, MM was maintained on tacrolimus monotherapy with trough levels of less than 6 ng/ml. Protocol biopsies at 8 and 24 weeks showed no signs of rejection. At 3 years, MM was in a stable clinical condition, receiving tacrolimus 2 mg BD with trough levels of 4–5 ng/ml as her sole maintenance immunosuppression.

The second patient, CA, a 47 year-old man, received a fully-mismatched kidney from a 40 year-old living, unrelated male donor. CA was treated with 7.1 × 10^6^ donor-derived M regs/kg seven days prior to transplantation under cover of 2 mg/kg/d azathioprine. Treatment with tacrolimus and steroids began the time of transplantation. Protocol biopsies at 8, 24 and 52 weeks showed no signs of rejection. At 3 years post-transplantation, CA had stable renal function and was being maintained with sustained-release tacrolimus 5 mg OD with a trough tacrolimus level of 2.7 ng/ml.

Minimising the maintenance immunosuppression of renal transplant recipients to tacrolimus monotherapy is not recommended by the Kidney Disease Improving Global Outcomes (KDIGO) guidelines, which advise the combination of a calcineurin inhibitor and an antiproliferative agent, with or without corticosteroids [61]. Nevertheless, in practice, patients receiving tacrolimus monotherapy are not uncommon and use of Alemtuzumab induction as a means of establishing patients on maintenance tacrolimus monotherapy has stimulated much recent interest. So, should we be surprised by the clinical outcomes of MM and CA? Although, we must be cautious in our interpretation of these two case studies, there are intriguing aspects of MM and CA’s histories that deserve particular mention: Both patients were minimised to *low-dose* tacrolimus monotherapy; this reduction in tacrolimus dosing was made relatively early after transplantation; neither MM nor CA received conventional induction therapy; and, both MM and CA developed a peripheral blood biomarker profile converging upon the IOT-RISET signature of tolerance [62].

What constitutes *low-dose* tacrolimus therapy? Definitions of low-dose and standard-dose tacrolimus treatment are largely a matter of convention. The *Symphony Study*, which assessed whether a mycophenolate mofetil (MMF)-based regimen allows minimisation of adjunct immunosuppression, incorporated a low-dose tacrolimus arm in which 75% of patients had trough tacrolimus levels of 4.3-10.0 ng/mL [63]. By this standard, MM and CA were treated with very low-dose tacrolimus, since both patients registered drug levels in the lowest 12.5 percentile of this range [64]. Unlike subjects in the Symphony Study, patients MM and CA were not given Dacluzimab induction or maintenance MMF therapy. Viewed in this context, the fact that both MM and CA remain rejection-free and with stable graft function at >4 years post-transplantation is an encouraging outcome.

**Figure 6:**
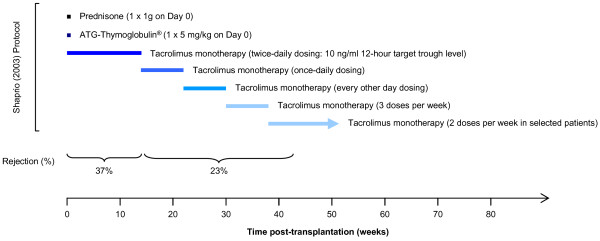
**Summary of a tacrolimus-minimisation study conducted by Shapiro et al.[**[65]**].** This clinical trial remains a benchmark study of minimised tacrolimus monotherapy in renal transplant recipients. 150 patients were treated with ATG induction therapy and bolus prednisone before being maintained on tacrolimus monotherapy. Over several months, tacrolimus was weaned in a stepwise fashion from 113 patients. The acute rejection rate prior to weaning was 37% and the acute rejection rate during weaning was 23%.

Is low-dose tacrolimus monotherapy difficult to achieve in renal transplant recipients? Shapiro’s 2003 study remains a benchmark trial of minimised tacrolimus monotherapy in renal transplant recipients [65]. 150 patients were treated with 5 mg/kg ATG and bolus prednisone as an induction therapy, and were subsequently maintained tacrolimus monotherapy, which was minimised in a step-wise fashion over many months (Figure 6). Under this regimen, 37% of patients underwent acute rejection prior to minimisation of tacrolimus dosing. 113 patients were then selected to undergo tacrolimus weaning: These patients were followed-up for a mean of 11 ± 5.4 months, during which time 23% of patients underwent acute rejection. Other studies with the aim of establishing renal transplant patients on tacrolimus monotherapy after ATG induction achieved similar outcomes [66]. More recently, Alemtuzumab induction with tacrolimus monotherapy has been used with some success [67]. Margreiter *et al*. reported a 20% 1-year biopsy-proven acute rejection rate in patients undergoing Alemtuzumab induction, followed by tacrolimus monotherapy with trough drug levels of 8–12 ng/ml for 6 months, reduced to 5–8 ng/ml thereafter [68]. More impressively, Chan and colleagues observed an 89.9% 2-year rejection-free renal allograft survival rate using Alemtuzumab and tacrolimus monotherapy with a target-range of 5–8 ng/ml [69]. Clearly, tacrolimus monotherapy can be achieved in renal transplant patients treated with a powerful induction agent, such as Alemtuzumab; nevertheless, it is still surprising that patients MM and CA, who were not treated with T cell-depleting monoclonal antibodies, were able to tolerate an early, fairly abrupt minimisation of immunosuppression.

A pattern of peripheral blood gene expression associated with a drug-free, tolerant state in renal transplant recipients has been defined by the IOT-RISET consortium [62]. To assess the immunological consequences of M reg therapy on patients MM and CA, expression of the ten most discriminatory gene markers of tolerance identified by the IOT-RISET group was quantified in serial blood samples taken from both patients [3]. Over the first year post-transplant, the pattern of expression of these markers gradually converged upon the IOT-RISET gene signature, suggesting that MM and CA might have achieved a degree of immunological regulation against their donors. To put this result into context, we have to consider the frequency of renal transplant recipients on CNI monotherapy whose gene expression profiles converge on the IOT-RISET signature: Of the 30 patients on CNI monotherapy included in the IOT-RISET cohort, all of whom were 6 to 9 years post-transplantation, only 5 displayed such a gene expression profile. Because the IOT-RISET study purposefully selected long-term, clinically stable patients, it is likely that 16% is an over-estimation of the frequency of CNI monotherapy patients in the general transplant population with the tolerance signature.

## Conclusions

Studies in animal models have shown the potential of immature DCs, MDSCs and regulatory macrophages to suppress T cell responses against alloantigen and to prolong allograft survival. This review has presented three very different strategies for myeloid cell therapy in solid organ transplantation. The particular cellular and molecular functions of different myeloid suppressor APCs might make each cell type more suitable for different clinical applications: Donor-derived M regs are to be used pre-operatively to induce donor-specific regulation, whereas recipient-derived suppressor cells and MDSCs have to be used peri- or post-operatively. Translation of myeloid suppressor APC therapy to the clinic is already underway. Several patients have now been treated with M regs in early-phase clinical trials and tolerogenic DCs are currently being tested in rheumatoid arthritis and diabetes studies. *The ONE Study* consortium is undertaking a clinical trial which should allow a side-by-side comparison of M regs and Tol-DC as adjunct immunosuppressive therapies in renal transplantation. The results of this study are awaited with great excitement.

## Abbreviations

Ag: Antigen; APC: Antigen presenting cell; CNI: Calcineurin inhibitor; IL: Interleukin; M-CSF: Monocyte colony stimulating factor; GM-CSF: Granulocyte monocyte colony stimulating factor; LN: lymph node; T reg: Regulatory T cell; MLR: Mixed lymphocyte reaction; NHP: Non-human primate.

## Competing interests

The authors declare that they have no competing interests.

## Authors' contributions

PR wrote the manuscript with assistance from EKG and JAH. All authors read and approved the final manuscript.

## References

[B1] Hutchinson JA Riquelme P Brem-Exner BG Schulze M Matthai M Renders L Kunzendorf U Geissler EK Fandrich F Transplant acceptance-inducing cells as an immune-conditioning therapy in renal transplantation Transpl Int 2008 21 728 741 10.1111/j.1432-2277.2008.00680.x 18573142

[B2] Hutchinson JA Brem-Exner BG Riquelme P Roelen D Schulze M Ivens K Grabensee B Witzke O Philipp T Renders L A cell-based approach to the minimization of immunosuppression in renal transplantation Transpl Int 2008 21 742 754 10.1111/j.1432-2277.2008.00692.x 18573141

[B3] Hutchinson JA Riquelme P Sawitzki B Tomiuk S Miqueu P Zuhayra M Oberg HH Pascher A Lutzen U Janssen U Cutting Edge: immunological consequences and trafficking of human regulatory macrophages administered to renal transplant recipients J Immunol 2011 187 2072 2078 10.4049/jimmunol.1100762 21804023

[B4] Harry RA Anderson AE Isaacs JD Hilkens CM Generation and characterisation of therapeutic tolerogenic dendritic cells for rheumatoid arthritis Ann Rheum Dis 2010 69 2042 2050 10.1136/ard.2009.126383 20551157 PMC3002758

[B5] Giannoukakis N Phillips B Finegold D Harnaha J Trucco M Phase I (safety) study of autologous tolerogenic dendritic cells in type 1 diabetic patients Diabetes Care 2011 34 2026 2032 10.2337/dc11-0472 21680720 PMC3161299

[B6] Geissler EK The ONE Study compares cell therapy products in organ transplantation: introduction to a review series on suppressive monocyte-derived cells Transplantation Research 2012 1 11 doi:10.1186/2047-1440-1-11 10.1186/2047-1440-1-11 PMC3561076 23369457

[B7] Hutchinson JA Riquelme P Geissler EK Human regulatory macrophages as a cell-based medicinal product Curr Opin Organ Transplant 2012 17 48 54 10.1097/MOT.0b013e32834ee64a 22186091

[B8] Beriou G Moreau A Cuturi MC Tolerogenic dendritic cells: applications for solid organ transplantation Curr Opin Organ Transplant 2012 17 42 47 10.1097/MOT.0b013e32834ee662 22227722

[B9] Thomson AW Tolerogenic dendritic cells: all present and correct? Am J Transplant 2010 10 214 219 10.1111/j.1600-6143.2009.02955.x 20055808 PMC2860031

[B10] Morelli AE Thomson AW Tolerogenic dendritic cells and the quest for transplant tolerance Nat Rev Immunol 2007 7 610 621 10.1038/nri2132 17627284

[B11] Amodio G Human tolerogenic DC-10: perspectives for clinical applications Transplantation Research 2012 1 14 doi:10.1186/2047-1440-1-14 10.1186/2047-1440-1-14 PMC3560992 23369527

[B12] Macedo C Immunoregulatory properties of rapamycin-conditioned monocyte-derived dendritic cells and their role in transplantation Transplantation Research 2012 1 16 doi:10.1186/2047-1440-1-16 10.1186/2047-1440-1-16 PMC3560974 23369601

[B13] Moreau A Cell therapy using tolerogenic dendritic cells in transplantation Transplantation Research 1 13 in press 10.1186/2047-1440-1-13 PMC3560975 23369513

[B14] Hutchinson JA Riquelme P Geissler EK Fandrich F Human regulatory macrophages Methods Mol Biol 2011 677 181 192 20941611 10.1007/978-1-60761-869-0_13

[B15] Riquelme P Govert F Geissler EK Fandrich F Hutchinson JA Human transplant acceptance-inducing cells suppress mitogen-stimulated T cell proliferation Transpl Immunol 2009 21 162 165 10.1016/j.trim.2009.03.004 19345264

[B16] Riquelme P Tomiuk S Kammler A Fandrich F Schlitt HJ Geissler EK 2012 Hutchinson JA: IFN-gamma-induced iNOS Expression in Mouse Regulatory Macrophages Prolongs Allograft Survival in Fully Immunocompetent Recipients. Mol Ther 10.1038/mt.2012.168 PMC3594012 22929659

[B17] Chauveau C Remy S Royer PJ Hill M Tanguy-Royer S Hubert FX Tesson L Brion R Beriou G Gregoire M Heme oxygenase-1 expression inhibits dendritic cell maturation and proinflammatory function but conserves IL-10 expression Blood 2005 106 1694 1702 10.1182/blood-2005-02-0494 15920011

[B18] Moreau A Hill M Thebault P Deschamps JY Chiffoleau E Chauveau C Moullier P Anegon I liot-Licht B Cuturi MC Tolerogenic dendritic cells actively inhibit T cells through heme oxygenase-1 in rodents and in nonhuman primates FASEB J 2009 23 3070 3077 10.1096/fj.08-128173 19420134

[B19] Hill M Thebault P Segovia M Louvet C Beriou G Tilly G Merieau E Anegon I Chiffoleau E Cuturi MC Cell therapy with autologous tolerogenic dendritic cells induces allograft tolerance through interferon-gamma and epstein-barr virus-induced gene 3 Am J Transplant 2011 11 2036 2045 10.1111/j.1600-6143.2011.03651.x 21794083

[B20] Peche H Trinite B Martinet B Cuturi MC Prolongation of heart allograft survival by immature dendritic cells generated from recipient type bone marrow progenitors Am J Transplant 2005 5 255 267 10.1111/j.1600-6143.2004.00683.x 15643985

[B21] Gregori S Tomasoni D Pacciani V Scirpoli M Battaglia M Magnani CF Hauben E Roncarolo MG Differentiation of type 1T regulatory cells (Tr1) by tolerogenic DC-10 requires the IL-10-dependent ILT4/HLA-G pathway Blood 2010 116 935 944 10.1182/blood-2009-07-234872 20448110

[B22] Turnquist HR Cardinal J Macedo C Rosborough BR Sumpter TL Geller DA Metes D Thomson AW mTOR and GSK-3 shape the CD4+ T-cell stimulatory and differentiation capacity of myeloid DCs after exposure to LPS Blood 2010 115 4758 4769 10.1182/blood-2009-10-251488 20335217 PMC2890188

[B23] Naranjo-Gomez M Raich-Regue D Onate C Grau-Lopez L Ramo-Tello C Pujol-Borrell R Martinez-Caceres E Borras FE Comparative study of clinical grade human tolerogenic dendritic cells J Transl Med 2011 9 89 10.1186/1479-5876-9-89 21658226 PMC3141500

[B24] Turnquist HR Fischer RT Thomson AW Pharmacological modification of dendritic cells to promote their tolerogenicity in transplantation Methods Mol Biol 2010 595 135 148 10.1007/978-1-60761-421-0_8 19941109

[B25] Taner T Hackstein H Wang Z Morelli AE Thomson AW Rapamycin-treated, alloantigen-pulsed host dendritic cells induce ag-specific T cell regulation and prolong graft survival Am J Transplant 2005 5 228 236 10.1046/j.1600-6143.2004.00673.x 15643982

[B26] Reichardt W Durr C Von ED Juttner E Gerlach UV Yamada M Smith B Negrin RS Zeiser R Impact of mammalian target of rapamycin inhibition on lymphoid homing and tolerogenic function of nanoparticle-labeled dendritic cells following allogeneic hematopoietic cell transplantation J Immunol 2008 181 4770 4779 18802080 10.4049/jimmunol.181.7.4770 PMC2881823

[B27] Greten TF Manns MP Korangy F Myeloid derived suppressor cells in human diseases Int Immunopharmacol 2011 11 802 807 10.1016/j.intimp.2011.01.003 21237299 PMC3478130

[B28] Dugast AS Haudebourg T Coulon F Heslan M Haspot F Poirier N de Vuillefroy SR Usal C Smit H Martinet B Myeloid-derived suppressor cells accumulate in kidney allograft tolerance and specifically suppress effector T cell expansion J Immunol 2008 180 7898 7906 18523253 10.4049/jimmunol.180.12.7898

[B29] De Wilde V Van RN Hill M Lebrun JF Lemaitre P Lhomme F Kubjak C Vokaer B Oldenhove G Charbonnier LM Endotoxin-induced myeloid-derived suppressor cells inhibit alloimmune responses via heme oxygenase-1 Am J Transplant 2009 9 2034 2047 10.1111/j.1600-6143.2009.02757.x 19681826

[B30] Obermajer N Muthuswamy R Lesnock J Edwards RP Kalinski P Positive feedback between PGE2 and COX2 redirects the differentiation of human dendritic cells toward stable myeloid-derived suppressor cells Blood 2011 118 5498 5505 10.1182/blood-2011-07-365825 21972293 PMC3217352

[B31] Li Z Pang Y Gara SK Achyut BR Heger C Goldsmith PK Lonning S Yang L Gr-1+CD11b+cells are responsible for tumor promoting effect of TGF-beta in breast cancer progression Int J Cancer 2012 131 2584 2595 10.1002/ijc.27572 22487809 PMC3433574

[B32] Srivastava MK Sinha P Clements VK Rodriguez P Ostrand-Rosenberg S Myeloid-derived suppressor cells inhibit T-cell activation by depleting cystine and cysteine Cancer Res 2010 70 68 77 10.1158/0008-5472.CAN-09-2587 20028852 PMC2805057

[B33] Bushell A Karim M Kingsley CI Wood KJ Pretransplant blood transfusion without additional immunotherapy generates CD25 + CD4+ regulatory T cells: a potential explanation for the blood-transfusion effect Transplantation 2003 76 449 455 10.1097/01.TP.0000083043.84630.99 12923427

[B34] Morelli AE Larregina AT Apoptotic cell-based therapies against transplant rejection: role of recipient’s dendritic cells Apoptosis 2010 15 1083 1097 10.1007/s10495-010-0469-9 20140521 PMC2929431

[B35] Steinman RM Turley S Mellman I Inaba K The induction of tolerance by dendritic cells that have captured apoptotic cells J Exp Med 2000 191 411 416 10.1084/jem.191.3.411 10662786 PMC2195815

[B36] Qiu CH Miyake Y Kaise H Kitamura H Ohara O Tanaka M Novel subset of CD8{alpha} + dendritic cells localized in the marginal zone is responsible for tolerance to cell-associated antigens J Immunol 2009 182 4127 4136 10.4049/jimmunol.0803364 19299710

[B37] Getts DR Turley DM Smith CE Harp CT McCarthy D Feeney EM Getts MT Martin AJ Luo X Terry RL Tolerance induced by apoptotic antigen-coupled leukocytes is induced by PD-L1+ and IL-10-producing splenic macrophages and maintained by T regulatory cells J Immunol 2011 187 2405 2417 10.4049/jimmunol.1004175 21821796 PMC3159828

[B38] Morelli AE Larregina AT Shufesky WJ Zahorchak AF Logar AJ Papworth GD Wang Z Watkins SC Falo LD Jr Thomson AW Internalization of circulating apoptotic cells by splenic marginal zone dendritic cells: dependence on complement receptors and effect on cytokine production Blood 2003 101 611 620 10.1182/blood-2002-06-1769 12393562

[B39] Quezada SA Fuller B Jarvinen LZ Gonzalez M Blazar BR Rudensky AY Strom TB Noelle RJ Mechanisms of donor-specific transfusion tolerance: preemptive induction of clonal T-cell exhaustion via indirect presentation Blood 2003 102 1920 1926 10.1182/blood-2003-02-0586 12750162

[B40] Pearson TC Madsen JC Larsen CP Morris PJ Wood KJ Induction of transplantation tolerance in adults using donor antigen and anti-CD4 monoclonal antibody Transplantation 1992 54 475 483 10.1097/00007890-199209000-00018 1384183

[B41] Ren G Su J Zhao X Zhang L Zhang J Roberts AI Zhang H Das G Shi Y Apoptotic cells induce immunosuppression through dendritic cells: critical roles of IFN-gamma and nitric oxide J Immunol 2008 181 3277 3284 18713999 10.4049/jimmunol.181.5.3277

[B42] Lu L Bonham CA Chambers FG Watkins SC Hoffman RA Simmons RL Thomson AW Induction of nitric oxide synthase in mouse dendritic cells by IFN-gamma, endotoxin, and interaction with allogeneic T cells: nitric oxide production is associated with dendritic cell apoptosis J Immunol 1996 157 3577 3586 8871658

[B43] Murray PJ Wynn TA Protective and pathogenic functions of macrophage subsets Nat Rev Immunol 2011 11 723 737 10.1038/nri3073 21997792 PMC3422549

[B44] Broichhausen C Riquelme P Geissler EK Hutchinson JA Regulatory macrophages as therapeutic targets and therapeutic agents in solid organ transplantation Curr Opin Organ Transplant 2012 17 332 342 22790067 10.1097/MOT.0b013e328355a979

[B45] Boros P Ochando JC Chen SH Bromberg JS Myeloid-derived suppressor cells: natural regulators for transplant tolerance Hum Immunol 2010 71 1061 1066 10.1016/j.humimm.2010.08.001 20705113 PMC3713408

[B46] Barnes MJ Powrie F Regulatory T cells reinforce intestinal homeostasis Immunity 2009 31 401 411 10.1016/j.immuni.2009.08.011 19766083

[B47] Day YJ Huang L Ye H Linden J Okusa MD Renal ischemia-reperfusion injury and adenosine 2A receptor-mediated tissue protection: role of macrophages Am J Physiol Renal Physiol 2005 288 F722 F731 15561971 10.1152/ajprenal.00378.2004

[B48] Jang HS Kim J Park YK Park KM Infiltrated macrophages contribute to recovery after ischemic injury but not to ischemic preconditioning in kidneys Transplantation 2008 85 447 455 10.1097/TP.0b013e318160f0d1 18301336

[B49] Gabrilovich DI Nagaraj S Myeloid-derived suppressor cells as regulators of the immune system Nat Rev Immunol 2009 9 162 174 10.1038/nri2506 19197294 PMC2828349

[B50] Obermajer N Generation of myeloid-derived suppressor cells using prostaglandin E2 Transplantation Research 2012 in press 10.1186/2047-1440-1-15 PMC3560989 23369567

[B51] Garcia MR Ledgerwood L Yang Y Xu J Lal G Burrell B Ma G Hashimoto D Li Y Boros P Monocytic suppressive cells mediate cardiovascular transplantation tolerance in mice J Clin Invest 2010 120 2486 2496 10.1172/JCI41628 20551515 PMC2898596

[B52] Divito SJ Wang Z Shufesky WJ Liu Q Tkacheva OA Montecalvo A Erdos G Larregina AT Morelli AE Endogenous dendritic cells mediate the effects of intravenously injected therapeutic immunosuppressive dendritic cells in transplantation Blood 2010 116 2694 2705 10.1182/blood-2009-10-251058 20576812 PMC2974582

[B53] Lan YY Wang Z Raimondi G Wu W Colvin BL De CA Thomson AW “Alternatively activated” dendritic cells preferentially secrete IL-10, expand Foxp3 + CD4+ T cells, and induce long-term organ allograft survival in combination with CTLA4-Ig J Immunol 2006 177 5868 5877 17056511 10.4049/jimmunol.177.9.5868

[B54] Beriou G Peche H Guillonneau C Merieau E Cuturi MC Donor-specific allograft tolerance by administration of recipient-derived immature dendritic cells and suboptimal immunosuppression Transplantation 2005 79 969 972 10.1097/01.TP.0000158277.50073.35 15849552

[B55] Dhodapkar MV Steinman RM Krasovsky J Munz C Bhardwaj N Antigen-specific inhibition of effector T cell function in humans after injection of immature dendritic cells J Exp Med 2001 193 233 238 10.1084/jem.193.2.233 11208863 PMC2193335

[B56] Hutchinson JA Govert F Riquelme P Brasen JH Brem-Exner BG Matthai M Schulze M Renders L Kunzendorf U Geissler EK Administration of donor-derived transplant acceptance-inducing cells to the recipients of renal transplants from deceased donors is technically feasible Clin Transplant 2009 23 140 145 10.1111/j.1399-0012.2008.00953.x 19200227

[B57] Hutchinson JA Roelen D Riquelme P Brem-Exner BG Witzke O Philipp T Matthai M Govert F Claas FH Westphal E Preoperative treatment of a presensitized kidney transplant recipient with donor-derived transplant acceptance-inducing cells Transpl Int 2008 21 808 813 10.1111/j.1432-2277.2008.00712.x 18573140

[B58] Warnecke G Hutchinson JA Riquelme P Kruse B Thissen S Avsar M Zehle G Steinkamp T Peters C Baumann R Postoperative intravenous infusion of donor-derived transplant acceptance-inducing cells as an adjunct immunosuppressive therapy in a porcine pulmonary allograft model Transpl Int 2009 22 332 341 10.1111/j.1432-2277.2008.00778.x 18954376

[B59] Tiurbe G Matuschek A Kammerer U Schneider M Thiede A Ulrichs K Otto C Inhibitory effects of rat bone marrow-derived dendritic cells on naive and alloantigen-specific CD4+ T cells: a comparison between dendritic cells generated with GM-CSF plus IL-4 and dendritic cells generated with GM-CSF plus IL-10 BMC Res Notes 2009 2 12 10.1186/1756-0500-2-12 19166583 PMC2639598

[B60] Chou HS Hsieh CC Charles R Wang L Wagner T Fung JJ Qian S Lu LL Myeloid-derived suppressor cells protect islet transplants by B7-H1 mediated enhancement of T regulatory cells Transplantation 2012 93 272 282 10.1097/TP.0b013e31823ffd39 22179405 PMC3267010

[B61] Kidney D Improving Global Outcomes (KDIGO) Transplant Work Group. KDIGO clinical practice guideline for the care of kidney transplant recipients Am J Transplant 2009 9 Suppl 3 S1 S155 10.1111/j.1600-6143.2009.02834.x 19845597

[B62] Sagoo P Perucha E Sawitzki B Tomiuk S Stephens DA Miqueu P Chapman S Craciun L Sergeant R Brouard S Development of a cross-platform biomarker signature to detect renal transplant tolerance in humans J Clin Invest 2010 120 1848 1861 10.1172/JCI39922 20501943 PMC2877932

[B63] Ekberg H Tedesco-Silva H Demirbas A Vitko S Nashan B Gurkan A Margreiter R Hugo C Grinyo JM Frei U Reduced exposure to calcineurin inhibitors in renal transplantation N Engl J Med 2007 357 2562 2575 10.1056/NEJMoa067411 18094377

[B64] Ekberg H Mamelok RD Pearson TC Vincenti F Tedesco-Silva H Daloze P The challenge of achieving target drug concentrations in clinical trials: experience from the Symphony study Transplantation 2009 87 1360 1366 10.1097/TP.0b013e3181a23cb2 19424036

[B65] Shapiro R Jordan ML Basu A Scantlebury V Potdar S Tan HP Gray EA Randhawa PS Murase N Zeevi A Kidney transplantation under a tolerogenic regimen of recipient pretreatment and low-dose postoperative immunosuppression with subsequent weaning Ann Surg 2003 238 520 525 14530723 10.1097/01.sla.0000089853.11184.53 PMC1360110

[B66] Tan HP Kaczorowski D Basu A McCauley J Marcos A Donaldson J Unruh M Randhawa P Zeevi A Shapiro R Steroid-free tacrolimus monotherapy after pretransplantation thymoglobulin or Campath and laparoscopy in living donor renal transplantation Transplant Proc 2005 37 4235 4240 10.1016/j.transproceed.2005.10.020 16387087

[B67] Tan HP Donaldson J Basu A Unruh M Randhawa P Sharma V Morgan C McCauley J Wu C Shah N Two hundred living donor kidney transplantations under alemtuzumab induction and tacrolimus monotherapy: 3-year follow-up Am J Transplant 2009 9 355 366 10.1111/j.1600-6143.2008.02492.x 19120078

[B68] Margreiter R Klempnauer J Neuhaus P Muehlbacher F Boesmueller C Calne RY Alemtuzumab (Campath-1 H) and tacrolimus monotherapy after renal transplantation: results of a prospective randomized trial Am J Transplant 2008 8 1480 1485 10.1111/j.1600-6143.2008.02273.x 18510632

[B69] Chan K Taube D Roufosse C Cook T Brookes P Goodall D Galliford J Cairns T Dorling A Duncan N Kidney transplantation with minimized maintenance: alemtuzumab induction with tacrolimus monotherapy-an open label, randomized trial Transplantation 2011 92 774 780 10.1097/TP.0b013e31822ca7ca 21836540

